# Mechanism and treatment of secondary glaucoma after corneal transplantation: a review

**DOI:** 10.3389/fopht.2024.1361704

**Published:** 2024-05-08

**Authors:** Yumeng Lin, Qiaoyin Gou, Ping Yu, Zhengfang Wu, Liuzhi Zeng, Haoran Chen

**Affiliations:** ^1^ Eye School of Chengdu University of Traditional Chinese Medicine, Chengdu, China; ^2^ Key Laboratory of Sichuan Province Ophthalmopathy Prevention & Cure and Visual Function Protection with Traditional Chinese Medicine (TCM) Laboratory, Chengdu, China; ^3^ Retinal Image Technology and Chronic Vascular Disease Prevention & Control and Collaborative Innovation Center, Chengdu, China; ^4^ Department of Ophthalmology, Chengdu First People’s Hospital, Chengdu, China; ^5^ Science Education Department, Chengdu Xinhua Hospital Affiliated to North Sichuan Medical College, Chengdu, China

**Keywords:** glaucoma, pathogenesis, treatment, corneal transplantation, eye

## Abstract

Corneal transplantation is a common treatment for corneal diseases. Secondary glaucoma after corneal transplantation is the second leading cause of failure of keratoplasty. This article reviews the mechanism and treatment of secondary glaucoma after corneal transplantation.

## Introduction

Keratopathy is one of the major blinding eye diseases nowadays and is the second leading blinding eye disease after cataract in China. At present, there are about 4 million patients with blindness caused by keratopathy in China, and more than 100,000 new cases are added each year (1). Corneal transplantation is the most effective treatment to restore vision or control keratopathy in the affected eye.

Common keratoplasty includes lamellar keratoplasty (LK), penetrating keratoplasty (PK), endothelial keratoplasty (EK), femtosecond laser assisted keratoplasty (FSL), and keratoprosthesis implantation (KPro). LK includes anterior lamellar keratoplasty (ALK) and posterior lamellar keratoplasty (PLK). EK includes deep lamellar endothelial keratoplasty (DLEK), Descemets stripping endothelial keratoplasty (DSEK), and Descemets membrane endothelial keratoplasty (DMEK).

In China, the indications for keratoplasty are mostly corneal infection and chemical burns, while in Western countries, the indications for keratoplasty are mainly keratoconus, bullous keratopathy, and corneal dystrophy. Since the early 20th century, keratoplasty has been first reported by Eduaed Zirm, and since then a variety of surgical procedures such as LK, EK, DSEK, DMEK, and DLEK have emerged successively, and keratoplasty has been widely used in clinical practice and has been greatly developed (2). Corneal transplantation is transparent tissue without blood vessels and lymphatic vessels, so it is not easily recognized by the immune system of the human body and is in the position of “immune privilege”. Keratoplasty is a relatively high success rate in all human tissue or organ transplantation surgeries.

Common complications after keratoplasty can be divided into early complications and late complications. Early postoperative complications include: 1. Poor postoperative corneal healing; 2. Interlamellar effusion and hematocele may occur after LK; 3. Graft detachment after EK; 4. Recurrence of primary infection or postoperative infection; 5. Secondary glaucoma. Late complications include: 1. Immune rejection; 2. Corneal graft turbidity. Secondary glaucoma is one of the most serious complications after keratoplasty and is second only to graft rejection leading to corneal graft failure (3).

It has been reported that the incidence of secondary glaucoma varies after different types of keratoplasty, with the incidence of early glaucoma after PK being 9-31%, the incidence of late postoperative glaucoma being 18-35%, and 2.8-6.5% after DMEK. Deep anterior lamellar keratoplasty (DALK) has a low incidence of glaucoma of 0-4.48% due to small inflammatory response, short duration of steroid eye drops use, and intact Descemet membrane preserving the angle structure (4–9). Glaucoma is a common complication due to anterior segment dysplasia or postoperative steroid use (5-9%). About half of the eyes of patients with Pitt’s abnormality develop glaucoma before or after surgery (10). According to Brittany Tsou et al., approximately 15 eyes (88.2%) of patients treated with Boston type I keratoprosthesis (KPro) surgery required recurrent glaucoma surgery (11).

## Pathogenesis of secondary glaucoma after corneal transplantation

The pathogenesis of secondary glaucoma after corneal transplantation is still unclear, but the possible causes and mechanisms have been reported in many literatures.

### Angle closure

It is mainly caused by trabecular meshwork collapse and peripheral anterior synechia (PAS).Mechanical collapse of the trabecular meshwork is proposed by Zimmerman et al., PK cuts off the Descemet’s membrane, so that the trabecular meshwork loses its anterior support, which in turn leads to poor aqueous outflow (12). Huber et al. found that patients with preoperative PAS have a higher proportion of secondary glaucoma after keratoplasty (13). Kirkness et al. found that repeated PK increases the risk of PAS formation, which in turn leads to angle closure to post penetrating keratoplasty glaucoma (PPKG) (14).

### Surgical factors

Improper intraoperative operation can cause damage to the trabecular meshwork, and the operation itself will also cause the release of prostaglandins, resulting in poor aqueous humor outflow and excessive production, followed by increased intraocular pressure. It has been reported that postoperative stress response and postoperative inflammation may lead to increased corneal thickness, which in turn leads to compression of the trabecular meshwork by anterior chamber angle stenosis interfering with the drainage of aqueous humor (15, 16). Whether these changes will combine with PAS to form permanent changes remains to be further studied (16).

### Postoperative glucocorticoid use

The mechanism of glucocorticoid-induced intraocular pressure elevation is not clear. Glucocorticoid induced upregulation of Myocilin, a gene encoding a 55-kda secretory protein, in trabecular meshwork cells and reorganization of the cytoskeletal and cross-linked actin networks, increasing the stiffness of the trabecular meshwork and inhibiting its contraction. Besides, cellular phagocytosis is weakened and extracellular matrix protein deposition is increased, leading to trabecular meshwork dysfunction, which leads to increased resistance to aqueous humor outflow (17, 18).

### Previous history of glaucoma

Huber et al. studied 160 patients with secondary glaucoma after PK surgery, 62 patients (38.7%) had previous history of glaucoma, which is the most important risk factor for secondary glaucoma after keratoplasty (13). Some scholars compared the incidence of PPKG between patients with glaucoma history and those without glaucoma history. The study showed that the incidence of PKGG was 59.4% in patients with preoperative glaucoma history and 14.6% in patients without glaucoma history (19).

### Pupillary block

Pupillary block is often caused early after DMEK due to excessive anterior chamber gas and aqueous circulation disorders (20). The mechanism of pupillary block is very similar between DMEK and DSEK. However, DMEK grafts without matrix require longer periods of bubble support and therefore result in a higher risk of pupillary block compared to DSEK grafts (21, 22).

### Lens condition

Aphakic eyes and intraocular lens eyes are important risk factors for secondary glaucoma after keratoplasty. Aphakic eyes and intraocular lens eyes have potential mechanical instability. The removal of lens causes the trabecular meshwork to lose the tension of zonules, weakens the support of ciliary body-lens support system for the trabecular meshwork posteriorly, decreases the aqueous humor fluency coefficient, and then causes the increase of intraocular pressure (8).

### Immune rejection

Onur et al. found that immune rejection caused a greater risk of PPKG. Rejection can cause increased prostaglandin secretion, a large number of inflammatory cell cellulose and iris pigment blocking the trabecular meshwork and aqueous humor external drainage channels, causing increased intraocular pressure (23).

## Treatment of secondary glaucoma after keratoplasty

The treatment of secondary glaucoma after keratoplasty mainly includes drug, laser and surgical treatment. The purpose of treatment is to reduce intraocular pressure and protect the optic nerve.

### Drug therapy

Secondary glaucoma after keratoplasty can be treated with ocular hypotensive drugs in the early stage. Drug therapy is the first-line treatment for secondary glaucoma after keratoplasty. Local ocular hypotensive drugs are mostly β-blockers such as timolol eye drops and carteolol eye drops, which can effectively treat increased intraocular pressure caused by shallow anterior chamber and angle closure after KPro surgery, but it may lead to decreased corneal epithelial barrier function, causing superficial punctate corneal epithelial lesions, making dry eye aggravated and corneal sensory loss (24–27).

Prostaglandin preparations can be used to treat symptoms of persistent elevated IOP after keratoplasty, but they may lead to anterior uveal inflammation, cystoid macular edema, increased rejection, and recurrence of some primary diseases (e.g., herpes simplex keratitis) (28–32).

The miotic pilocarpine increases the permeability of the blood-aqueous barrier in patients, thereby increasing the risk of graft rejection (33, 34). When IOP is poorly controlled, carbonic anhydrase inhibitors such as brinzolamide eye drops can be added in combination, but they may lead to graft endothelial decompensation, cystoid macular edema, or persistent inflammation (35–38). Oral carbonic anhydrase inhibitors cause serious hypokalemic side effects. Inhibition of carbonic anhydrase activity increases the excretion of NaCl and HCO_3_^-^ in the proximal convoluted tubules and increases the exchange of K^+^ -Na^+^ in the distal nephridium, resulting in low potassium (39, 40).

As an RHO kinase inhibitor, nesudil inhibits Rho kinase to dilate the connective tissue proximal to the trabecular meshwork and reduce superficial scleral venous pressure, thereby increasing aqueous outflow. Nesudil is a relatively new drug in the treatment of glaucoma and corneal edema, but studies have shown that it may cause transient vision loss (41).

In the treatment of intraocular pressure with combined drugs, if the conventional combined drugs fail to achieve the expected effect, oral ocular hypotensive drugs such as methazolamide can be added. The medication process should be followed up and reexamined to monitor the patient ‘s intraocular pressure and adjust the medication regimen in time. For patients with poor compliance and irregular reexamination, when their intraocular pressure is in a dangerous state value, emergency ocular hypotensive regimen should be used, local eye drops combined with oral drugs, and then mannitol 1 mg/kg intravenous drip should be added for treatment.

Perry et al. found that switching to 0.5% cyclosporine eye drops after keratoplasty reduced intraocular pressure and prevented the occurrence of rejection (42). For steroid-sensitive patients, glucocorticoid dosage could be gradually reduced or steroid drugs with less efficacy such as fluorometholone could be used instead of glucocorticoids in clinical practice, which could prevent rejection and reduce the risk of increased intraocular pressure.

### Laser therapy

#### Laser peripheral iridotomy

LPI removes a small amount of iris tissue in the periphery of the iris by laser to allow the posterior chamber aqueous humor to flow directly into the anterior chamber through this incision and relieve the obstruction of the anterior bulge of the peripheral iris and the anterior chamber angle due to pupillary block. It is not clear whether LPI must be performed only in the case of patients affected by pupillary block or if it may be adopted as a general surgical technique to facilitate aqueous humor flow from posterior to anterior chamber. Fu Ronghua et al. reported that intraocular pressure decreased in one of two patients with pupillary membrane closure after penetrating keratoplasty after YAG laser treatment, and intraocular pressure was well controlled in the other after trabeculectomy combined with mitomycin C (MMC). The study by Yin Wenhui et al. showed that intraocular pressure could be well controlled in most patients with pupillary block after keratoplasty who underwent laser peripheral iridectomy.

#### Argon laser peripheral iridoplasty

ALPI uses an argon ion laser to laser irradiate the iris root using a large spot, low energy, and long exposure time, and the iris matrix contracts at the burned site, which in turn opens the angle by physical traction. Dada et al. concluded that the main cause of advanced PPKG is adhesive angle closure, and the degree of angle closure is closely related to intraoperative operation, and they concluded that relaxed and atrophic iris can lead to a higher incidence of PAS formation, which can be prevented by iridoplasty (8).

#### Selective laser trabeculoplasty

Laser trabeculoplasty acts on the trabecular meshwork through YAG laser to realize the remodeling of trabecular meshwork structure and function, reduce the resistance of trabecular meshwork aqueous outflow, and finally achieve the reduction of intraocular pressure. Nakakura et al. reported a case of SLT after PK surgery, long-term use of latanoprost 6 months after surgery, and follow-up intraocular pressure up to 18 mmHg, but its long-term efficacy is poor (43).

### Operative therapy

#### Goniosynechialysis

GSL increases aqueous humor outflow by separating iris tissue adhering to the angle and reopening the angle, thereby reducing intraocular pressure (44). It has been documented that peripheral iris adhesions are an important cause of secondary acute angle-closure glaucoma after Descemet’s stripping automated endothelial keratoplasty (DSAEK). Severe corneal graft edema in patients can lead to unclear vision by physicians, and endoscopic fiberoptic fibers and camera probes are mostly used to visualize the attached iris tissue in clinical practice. Physicians can gently pull apart the iris tissue with miniature forceps and successfully complete 270° synechiolysis to restore trabecular function, and no corneal rejection has been found after surgery (45). However, some scholars believe that GSL can lead to uveitis, hyphema and iridodialysis and other side effects (46).

#### Peripheral iridotomy

A small amount of iris tissue is removed from the periphery of the iris in patients, and posterior chamber aqueous humor can flow directly into the anterior chamber through this incision. Some scholars have found through studies that 12.5% of patients have persistent intraocular pressure elevation of more than 30 mmHg after DMEK, but patients do not have any other symptoms, so peripheral iridectomy is recommended in such patients. It should be noted that peripheral iridectomy may be obstructed by bubbles, foreign bodies, or heme (47). The principle of PI is similar to that of LPI, but different from LPI, which uses laser to punch the iris tissue, PI surgically resects part of the iris tissue. For patients who cannot be operated by laser for various reasons, such as hard iris tissue, surgical resection can be used.

#### Trabeculectomy

TRA is a new aqueous humor drainage channel established at the limbus to drain aqueous humor from the anterior chamber to the subconjunctival space, and then absorbed by the surrounding tissues, which is a traditional surgical approach for the treatment of secondary glaucoma. TRA combined with mitomycin C (MMC) can successfully reduce intraocular pressure, but there is a certain probability of corneal transplantation failure and complications. Lin Yongfeng et al. found that TRA combined with MMC can form effective filtering blebs, which have a good long-term effect in reducing intraocular pressure and can effectively improve visual acuity. It should be noted that the probability of transplantation failure caused by corneal scars and local tissue fibrosis is 12-18% (48, 49). MMC has not increased the probability of corneal transplantation failure, but MMC has the possibility of causing scleral necrosis and chronic bubble leakage (50, 51). Superficial anterior chamber is a common complication of filtering surgery, and studies have shown that the incidence of shallow anterior chamber after trabeculectomy combined with MMC is as high as 32% (52).

#### Non-perforating deep sclerectomy

Deep sclera was removed, the inner wall of Schlemm ‘s canal and clinical canal tissue were avulsed, and aqueous humor was drained from the anterior chamber into the subconjunctival space, which is the treatment of choice for PPKG without PAS (53). One scholar compared the efficacy of NPDS and TRA in the treatment of PPKG and found that the success rates at 1 and 5 years were 76% and 44% in the NPDS group and 69% and 49% in the trabeculectomy group, respectively (54). NPDS was as effective as trabeculectomy in controlling intraocular pressure, but its long-term survival rate of the graft after surgery was better (53). Another study using NPDS after DSAEK for glaucoma came to the same conclusion (55).

#### Glaucoma drainage devices

The GDD consists of a silicone tube that drains aqueous humor from the anterior or posterior chamber to a drainage disc located in the equatorial region of the eyeball. Several weeks after implantation, a fibrous capsule forms around the drainage disc, and aqueous humor accumulates in the potential gap between the disc and the peripheral capsule. Then it diffuses passively through the capsule wall into periocular capillaries and lymphatic vessels, and the fibrous capsule has a major limiting effect on the outflow of aqueous humor in GDD. GDD, also known as an aqueous humor shunt device, is a small reconstructive surgical device that can be solid or made from a tube fixed to the endplate. A drainage orifice was created surgically and the implant placed correctly on it. All implants were designed to reduce intraocular pressure by increasing intraocular fluid outflow. There are many types of GDD, common ones are: Molteno single and double plate implants, Baerveldt drainage implants, Schocket implants, Ex-Press R50 implants, Ahmed glaucoma drainage valves(AGD), Krypton implants, And the latest iStent, iStent inject, Hydrus, CyPass, XEN and InnFocus (56).GDD is indicated for patients with significant anterior segment inflammation or severe bulbar conjunctival scarring. When combined with medical therapy, the success rate of intraocular pressure control ranges from 62% to 96% (57, 58). When combined with medical therapy, the success rate of IOP control ranges from 62% to 96% [68, 69]. GDD tends to be more successful in controlling IOP, but the rate of graft failure is also higher compared with trabeculectomy (59–61). Almousa et al. reported that the success rate of AGD implantation after secondary glaucoma in 59 high-risk keratoplasty patients was 75.8%, the survival rate of patients was 87% after 1 year, and the survival rate of patients decreased to 47% after 5 years (62). Li Navy et al. showed that AGD implantation reduced the incidence of shallow anterior chamber more than TRA. Studies have demonstrated that GDD can effectively reduce intraocular pressure and is an important method for the treatment of refractory glaucoma. It should be noted that mechanical corneal endothelial injury, reflux of inflammatory cells into the anterior chamber, and drainage valve implantation can affect the blood-eye barrier of patients and then change the protein content of aqueous humor, resulting in an increased rate of corneal transplantation failure in patients (59, 63–66). Some scholars have proposed that posterior chamber implantation for GDD can reduce the risk of transplantation failure (57).

#### Transscleral ciliary photocoagulation

The principle of TSCPC is that the laser destroys the structure of the ciliary body, thereby reducing aqueous humor production, and is suitable for patients with poor visual acuity, having undergone multiple ocular surgeries, and severe conjunctival scarring. Rivier et al. reported 18 Boston Keratoprothesis (B-KPro) eyes treated with TSCPC for secondary glaucoma, of which 12 eyes had intraocular pressure reduced to less than 20 mmHg, suggesting that TSCPC may be particularly suitable for B-KPro eyes (67). Alejandro et al. reported that the mean preoperative intraocular pressure in 16 PPKG patients was 31.5 mmHg, which decreased to 17.5 mmHg after the first application of diode laser, with an overall reduction of 14.0 mmHg (55.5% reduction) (68). Destructive surgery of the ciliary body has a high probability of complications such as corneal graft failure, hypotony, visual loss, and ocular atrophy, so this surgery is a treatment modality after ineffective use of other interventions (3).

#### Micropulse transscleral cyclophotocoagulation

MP-TSCPC is a disruptive surgery that uses continuous diode laser irradiation of the ciliary body to reduce the generation of aqueous humor (56). It is commonly used in the treatment of refractory glaucoma. And reduce aqueous humor production by destroying the non-pigmented epithelium of the ciliary body. MP-TSCPC is a form of cyclophotocoagulation in which the transmitted micropulse wave is set to an alternating cycle of “ON” and “OFF”, so that the laser energy acts on the ciliary body not in a continuous manner, but in an intermittent and periodic manner. In a short energy pulse (“ON” period), the laser energy acts on the ciliary epithelium. In a pause period in which no energy is delivered (“OFF” period), the adjacent tissue is cooled, thus protecting it from changes caused by high temperature (69). Therefore, MP-TSCPC has a satisfactory efficiency of lowering intraocular pressure, while producing fewer adverse effects (70–74).

Mihail Zemba et al. observed 29 glaucoma patients treated with MP-TSCPC after PK surgery for up to one year and found that the success rate of MP-TSCPC in lowering intraocular pressure after 12 months was 76%, resulting in corneal graft failure due to severe postoperative inflammatory response in only one patient (69). MP-TSCPC is a safe and effective surgical method for patients after keratoplasty to achieve ideal intraocular pressure control and success rate, while minimizing complications and graft failure rate.

#### Minimally invasive glaucoma surgery

Compared with traditional glaucoma surgery, MIGS has the advantage that its placement device is smaller and avoids damage to the corneal endothelium of patients. At present, only a single case report has described the role of MIGS implant device in glaucoma after keratoplasty. Rahmania et al. reported a patient with secondary glaucoma after keratoplasty treated with Xen45 gel stent who reported a 70.5% decrease in mean intraocular pressure and only one transient intraocular pressure increase at 7 days after surgery (75).

In conclusion, MIGS may be a new option for the treatment of glaucoma after keratoplasty. Due to the limited clinical data, its efficacy and safety need to be further studied.

## Conclusion

Secondary glaucoma after keratoplasty can be treated with drugs, laser or surgery, and uncontrolled intraocular pressure elevation will lead to corneal graft and optic nerve damage in patients. Some scholars have proposed that the transplanted corneal endothelium is more vulnerable to damage than its own corneal endothelium (76). Therefore, early detection, control of intraocular pressure, and timely treatment are essential to promote the survival of grafts and protect vision in patients (77).

Drugs are the first-line treatment for secondary glaucoma after keratoplasty. In the face of patients with different conditions, we should individualize the choice of treatment options. Surgical treatment is suitable for patients whose intraocular pressure remains uncontrolled after the use of the maximum dose of local anti-glaucoma drugs.

The field of glaucoma surgery is undergoing a profound change from traditional glaucoma surgery to MIGS surgery. MIGS surgery has many advantages such as less tissue destruction, less postoperative complications, safety and effectiveness, and is the development trend of glaucoma surgery technology in the future. MIGS provides a new option in the treatment of secondary glaucoma after keratoplasty. However, there are few literatures on the application of MIGS in the treatment of secondary glaucoma after keratoplasty, so more clinical data are needed to further study its efficacy and safety.

## Author contributions

YL: Writing – original draft. GQ: Writing – original draft. PY: Writing – original draft. ZW: Writing – original draft. LZ: Writing – review & editing. HC: Funding acquisition, Writing – review & editing.
